# Supplementary material for: Availability of mRNA Obtained from Peripheral Blood Mononuclear Cells for Testing Mutation Consequences in Dystrophic Epidermolysis Bullosa
Source: Int J Mol Sci. 2021 Dec 13;22(24):13369. doi: 10.3390/ijms222413369 (PMC8709150; doi:10.3390/ijms222413369)
Supplement: Supplementary file 1 [file ijms-22-13369-s001.zip › ijms-1483080-supplementary.pdf]

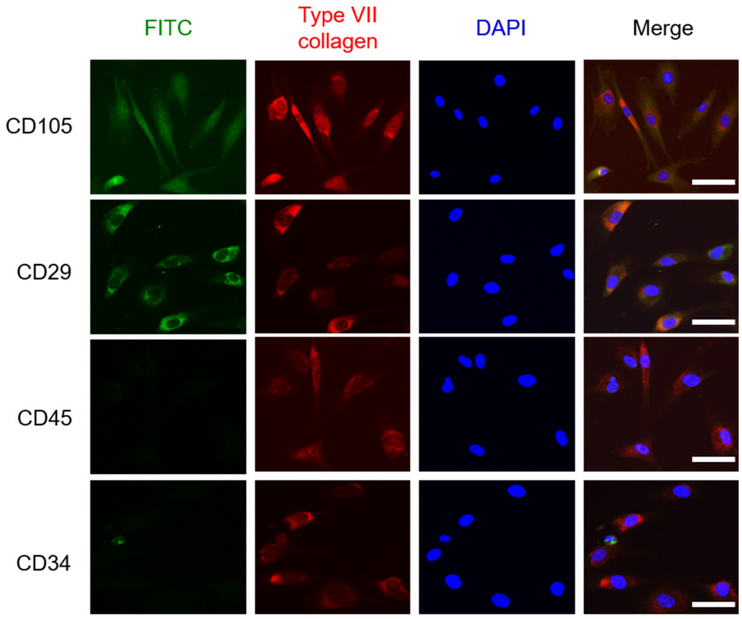

Figure S1. Immunophenotyping of cultured mesenchymal stem cells. Immunofluorescence staining for lineage markers CD105, CD29, CD45, and CD34 (green); type VII collagen (red); and nuclear component (blue). Scale bar, 100 μm. DAPI, 4',6-diamidino-2-phenylindole; FITC, fluorescein isothiocyanate.
